# 

**Published:** 1884-03

**Authors:** 


CONTENTS.

| | Page. | | Page. |
|---|---|---|---|
| Bright's Disease | 39 | Tubercle | 45 |
| Mother and Infant | 40 | Spitting Blood | 46 |
| Signs of Disease | 40 | Imperfect Breathing | 46 |
| Water on the Brain | 40 | Impure Blood | 47 |
| Condition of the Bowels | 41 | Converting Food into Blood | 49 |
| Crying | 42 | Pure Air | 50 |
| Consumption | 43 | Cough | 55 |
| Croup | 43 | The Pulse | 56 |
| Bronchitis | 44 | Accelerated Breathing | 57 |

